# Supplementary material for: PIP4K2B is mechanoresponsive and controls heterochromatin-driven nuclear softening through UHRF1
Source: Nat Commun. 2023 Mar 14;14:1432. doi: 10.1038/s41467-023-37064-0 (PMC10015053; doi:10.1038/s41467-023-37064-0)
Supplement: Supplementary file 1 — Supplemetary Informations [file 41467_2023_37064_MOESM1_ESM.pdf]

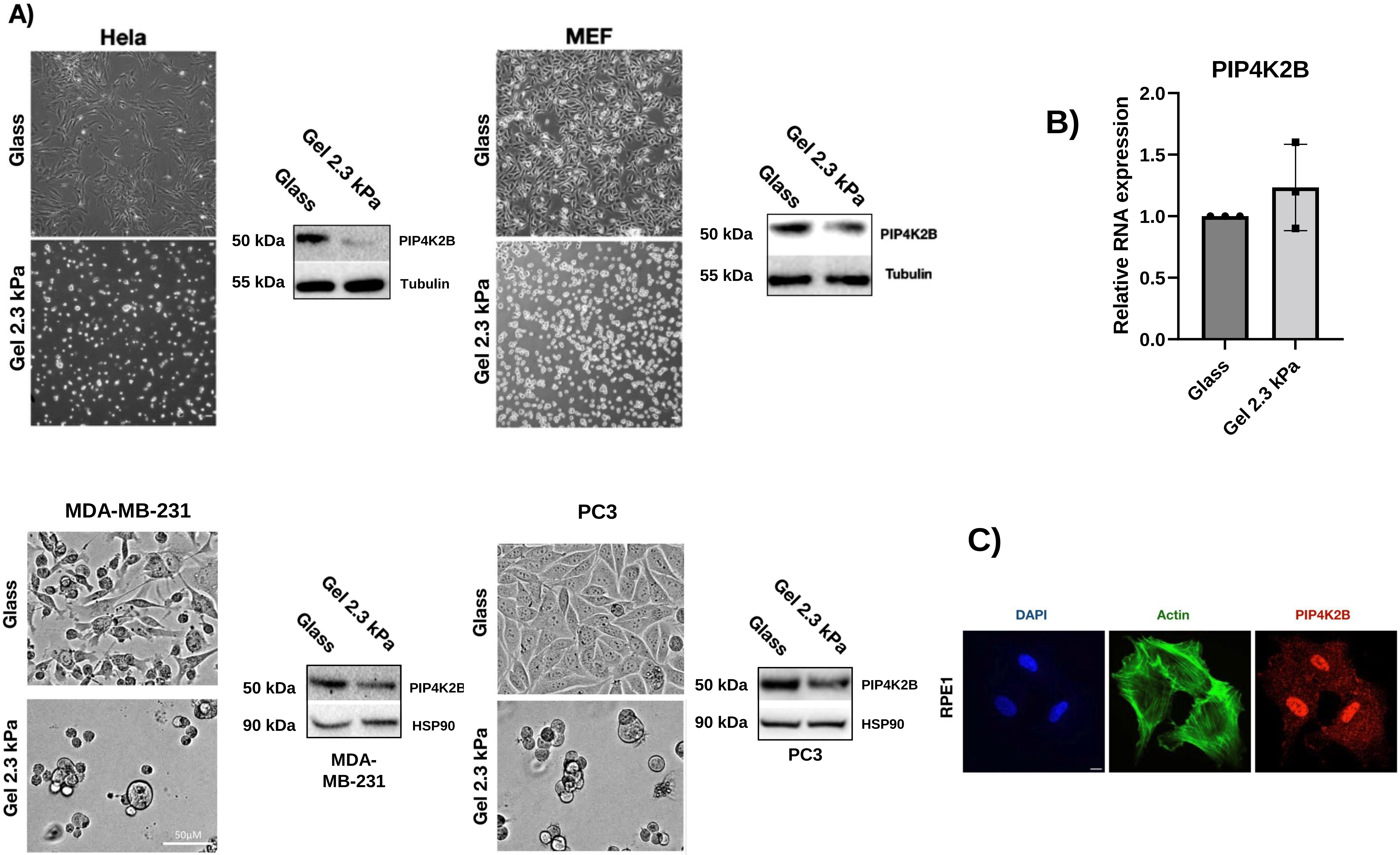

A)
Hela
Glass
Gel 2.3 kPa
Glass
Gel 2.3 kPa
50 kDa
PIP4K2B
55 kDa
Tubulin
MEF
Glass
Gel 2.3 kPa
Glass
Gel 2.3 kPa
50 kDa
PIP4K2B
55 kDa
Tubulin
B)
PIP4K2B
Relative RNA expression
2.0
1.5
1.0
0.5
0.0
Glass
Gel 2.3 kPa
MDA-MB-231
Glass
Gel 2.3 kPa
50µM
Glass
Gel 2.3 kPa
50 kDa
PIP4K2B
90 kDa
HSP90
MDA-MB-231
PC3
Glass
Gel 2.3 kPa
Glass
Gel 2.3 kPa
50 kDa
PIP4K2B
90 kDa
HSP90
PC3
C)
DAPI
Actin
PIP4K2B
RPE1

Supplementary Figure 1. PIP4K2B levels decrease in cells seeded on soft susbtrates. A) Hela, MEF, PC3 and MDA-MB-231 cells were grown on FN-coated glass (stiff) or elastic surface (Gel 2.3kPa, soft) for 24h, 24, 36h and 48h respectively, then lysed (scale bar = 20µm). Protein lysates were then immunoblotted to analyse PIP4K2B expression. β-Tubulin or HSP90 were used as loading control. B) RT-qPCR analysis of mRNA levels in hTERT_RPE1 cells grown as in A). Data are representative of n=3 independent experiments. GAPDH was used as housekeeping gene. Data are shown as Log2FoldChange and as bar charts +/- standard deviation. C) Immunofluorescence staining of hTERT_RPE1 cells for PIP4K2B, Actin (Phalloidin) and nuclei (DAPI) (scale bar = 10µm). Data are representative of n=3 experiments.

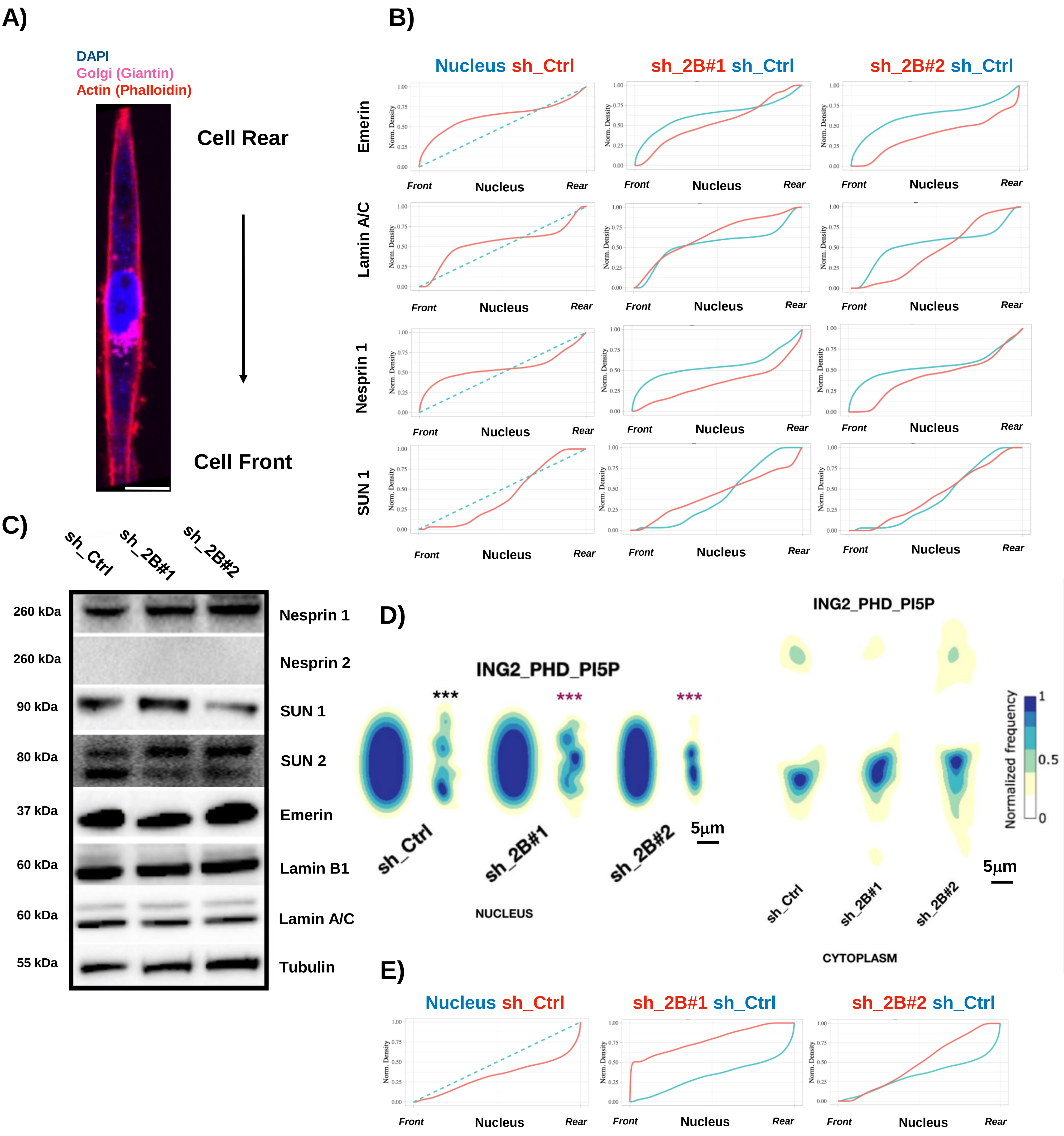
A)
DAPI
Golgi (Giantin)
Actin (Phalloidin)
Cell Rear
Cell Front
B)
Nucleus sh_Ctrl
sh_2B#1 sh_Ctrl
sh_2B#2 sh_Ctrl
Emerin
Lamin A/C
Nesprin 1
SUN 1
Norm. Density
Front
Nucleus
Rear
C)
sh_Ctrl
sh_2B#1
sh_2B#2
260 kDa
260 kDa
90 kDa
80 kDa
37 kDa
60 kDa
60 kDa
55 kDa
Nesprin 1
Nesprin 2
SUN 1
SUN 2
Emerin
Lamin B1
Lamin A/C
Tubulin
D)
ING2_PHD_PI5P
***
***
***
sh_Ctrl
sh_2B#1
sh_2B#2
5µm
NUCLEUS
ING2_PHD_PI5P
Normalized frequency
1
0.5
0
5µm
sh_Ctrl
sh_2B#1
sh_2B#2
CYTOPLASM
E)
Nucleus sh_Ctrl
sh_2B#1 sh_Ctrl
sh_2B#2 sh_Ctrl
Norm. Density
Front
Nucleus
Rear

Supplementary Figure 2. Depletion of PIP4K2B impacts on nuclear polarity. A) Immunofluorescence staining for Actin (Phalloidin), nucleus (DAPI) and Golgi (Giantin) in hTERT_RPE1 seeded on FN-coated coverslips displaying linear (10μm width) PLL-g-Peg patterns. Orientation of the cells (Front/Rear) was assessed considering Golgi localisation always at the front. Scale bar 10μm. B) Protein distribution plots of normalized density, corresponding to maps in Figure 2F (sh_Ctrl Emerin pVal =7.57x10-5, sh_2B#1 Emerin pVal=0.0356, sh_2B#2 Emerin pVal=0.000118; sh_Ctrl Lamin A/C pVal =0.00131, sh_2B#1 Lamin A/C pVal=0.000687, sh_2B#2 Lamin A/C pVal=0.000716; sh_Ctrl Nesprin1 pVal =0. 00314, sh_2B#1 Nesprin1 pVal=0. 00271, sh_2B#2 Nesprin1 pVal=0. 0188; sh_Ctrl SUN1 pVal =0. 0734, sh_2B#1 SUN1 pVal=0. 0610, sh_2B#2 SUN1 pVal=0. 704). C) Western Blotting showing expression of nuclear envelope proteins in hTERT_RPE1 cells transduced to silence PIP4K2B (sh_2B#1/#2), or with empty pLKO_1 vector as control (sh_Ctrl). D) Orientation maps of nuclear (left) and cytoplasmic (right) localisation of PI5P, detected using ING2_PHD-GFP probe. Cells were transduced to silence PIP4K2B (sh_2B#1/#2, n=36/51), or with empty pLKO_1 vector as control (sh_Ctrl, n=50) and data are representative of n=2 independent experiments (sh_Ctrl ING2=5.45x10-5; sh_2B#1 ING2=9.2x10-17; sh_2B#2 ING2=7.212x10-5). Scale bar 5μm. E) Protein distribution plots of normalized density, corresponding to maps in Supplementary Figure 2D. Statistical analysis was performed using two-sided Kolmogorov–Smirnov to test front versus back or two-sided Cramer-von Mises to test sh_Ctrl versus sh_PIP4K2B conditions for maps distribution reported in 1F with p values as *p < 0.05, **p < 0.01, ***p < 0.001. Black * indicates statistical analysis of the front vs rear accumulation, while purple * indicates statistical analysis of Ctrl cells vs PIP4K2B knock-down cells.

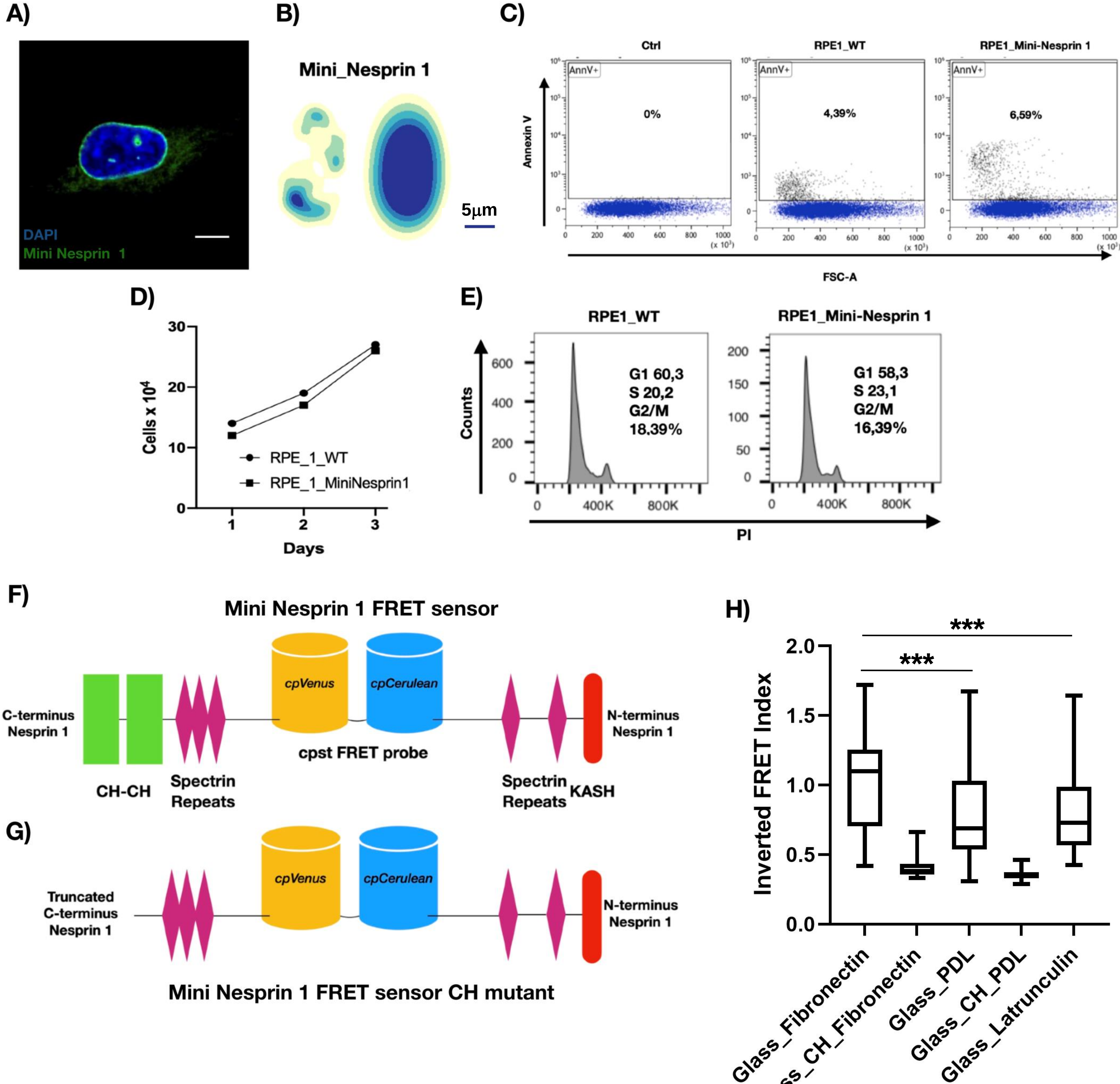
A)
DAPI
Mini Nesprin 1
B)
Mini_Nesprin 1
5µm
C)
Ctrl
RPE1_WT
RPE1_Mini-Nesprin 1
AnnV+
0%
4,39%
6,59%
Annexin V
FSC-A
D)
Cells x 10^4
RPE_1_WT
RPE_1_MiniNesprin1
Days
E)
RPE1_WT
RPE1_Mini-Nesprin 1
G1 60,3
S 20,2
G2/M
18.39%
G1 58,3
S 23,1
G2/M
16,39%
Counts
PI
F)
Mini Nesprin 1 FRET sensor
C-terminus Nesprin 1
cpVenus
cpCerulean
N-terminus Nesprin 1
cpst FRET probe
CH-CH
Spectrin Repeats
Spectrin Repeats
KASH
G)
Truncated C-terminus Nesprin 1
cpVenus
cpCerulean
N-terminus Nesprin 1
Mini Nesprin 1 FRET sensor CH mutant
H)
Inverted FRET Index
***
***
Glass_Fibronectin
Glass_CH_Fibronectin
Glass_PDL
Glass_CH_PDL
Glass_Latrunculin

Supplementary Figure 3. Development of a cpst-based FRET sensor to analyse nuclear envelope tensional state. A) Immunofluorescence staining for Mini Neprin 1 cpst-FRET sensor (green) and nucleus (DAPI) (scale bar = 10μm). Data are representative of n=3 experiments. B) Orientation maps of nuclear localisation of Mini Neprin 1 cpst-FRET sensor (n=15). Scale bar 5μm and data are representative of n=3 experiments. C) FACS based Annexin-V assay to assess apoptosis in hTERT_RPE1 cells wild type (RPE1_WT) or transfected to express Mini Neprin 1 cpst-FRET sensor (RPE_1 Mini_Nesprin 1). D) Cell proliferation assay performed in 24 well plates. 104 hTERT_RPE1 cells wild type (RPE1_WT) or transfected to express Mini Neprin 1 cpst-FRET sensor (RPE_1 Mini_Nesprin 1) were plated and then manually counted for 3 days. Data are representative of n=3 experiments. E) FACS based cell cycle analysis employing propidium iodide (PI) incorporation in the cells of hTERT_RPE1 cells wild type (RPE1_WT) or transfected to express Mini Neprin 1 cpst-FRET sensor (RPE_1 Mini_Nesprin 1). Data are representative of n=2 experiments. F)/G) Graphical representation of Mini Neprin 1 cpst-FRET sensor (top) and CH-CH mutant (bottom). H) Nuclear envelope (NE) tension analysis exploiting Mini Nesprin 1 cpst-FRET sensor wild-type version (WT, Glass Fibronectin n=20, Glass CH Fibronectin n=22) or CH-CH_mutant (CH, Glass_PDL n=25, Glass CH PDL n=24). hTERT_RPE1 cells expressing either WT or CH versions of the FRET sensor were seeded on FN-coated glass or soft surfaces (Gel 2.3 kPa). Cells on FN-coated glass were treated with Latrunculin (1μM, n=25) for 10 minutes and analysed. Data quantification is shown as boxplot chart representing inverted FRET index values (donor/acceptor, the higher the value, the higher the tension) and. Data shown derive from at least n=2 independent experiments. Statistical analyses were performed using unpaired two-tailed Student's t test with Welch's correction, with p values as ***p < 0.001. In boxplots: middle bars are medians, the rectangles span from the first to the third quartiles and bars extent from min to max values.

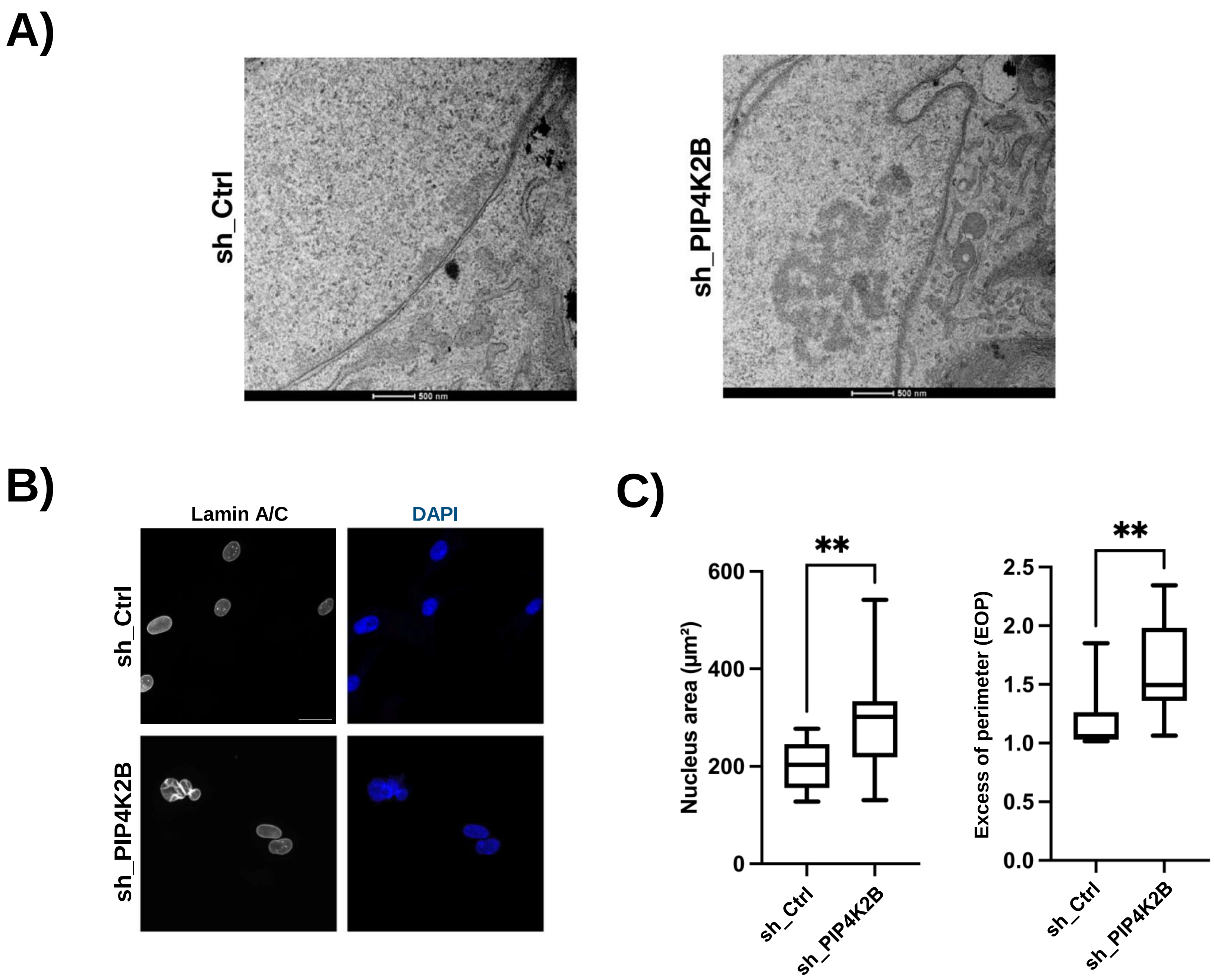

A)
sh_Ctrl
sh_PIP4K2B
500 nm
500 nm
B)
Lamin A/C
DAPI
sh_Ctrl
sh_PIP4K2B
C)
**
Nucleus area (μm²)
600
400
200
0
sh_Ctrl
sh_PIP4K2B
**
Excess of perimeter (EOP)
2.5
2.0
1.5
1.0
0.5
0.0
sh_Ctrl
sh_PIP4K2B

Supplementary Figure 4. Lack of PIP4K2B alters nuclear morphology. A) TEM images of sh_Ctrl or sh_PIP4K2B cells. Cropped images are presented in Figure 2 (scale bar = 500nm). B) and C) Immunofluorescence staining of Lamin A/C and DAPI of hTERT_RPE1 cells transduced to silence PIP4K2B (sh_2B, pVal 0.025 for nuclear area, pVal 0.027 for EOP), or with empty pLKO_1 vector as control (sh_Ctrl). Scale bar 30μm. Nuclear size and nuclear invaginations analyses is reported (see materials and methods). Statistical analyses were performed using unpaired two-tailed Student's t test with Welch's correction and derive from n=2 independent experiments with p values as $^{**}p < 0.01$. In boxplots: middle bars are medians, the rectangles span from the first to the third quartiles and bars extent from min to max values.

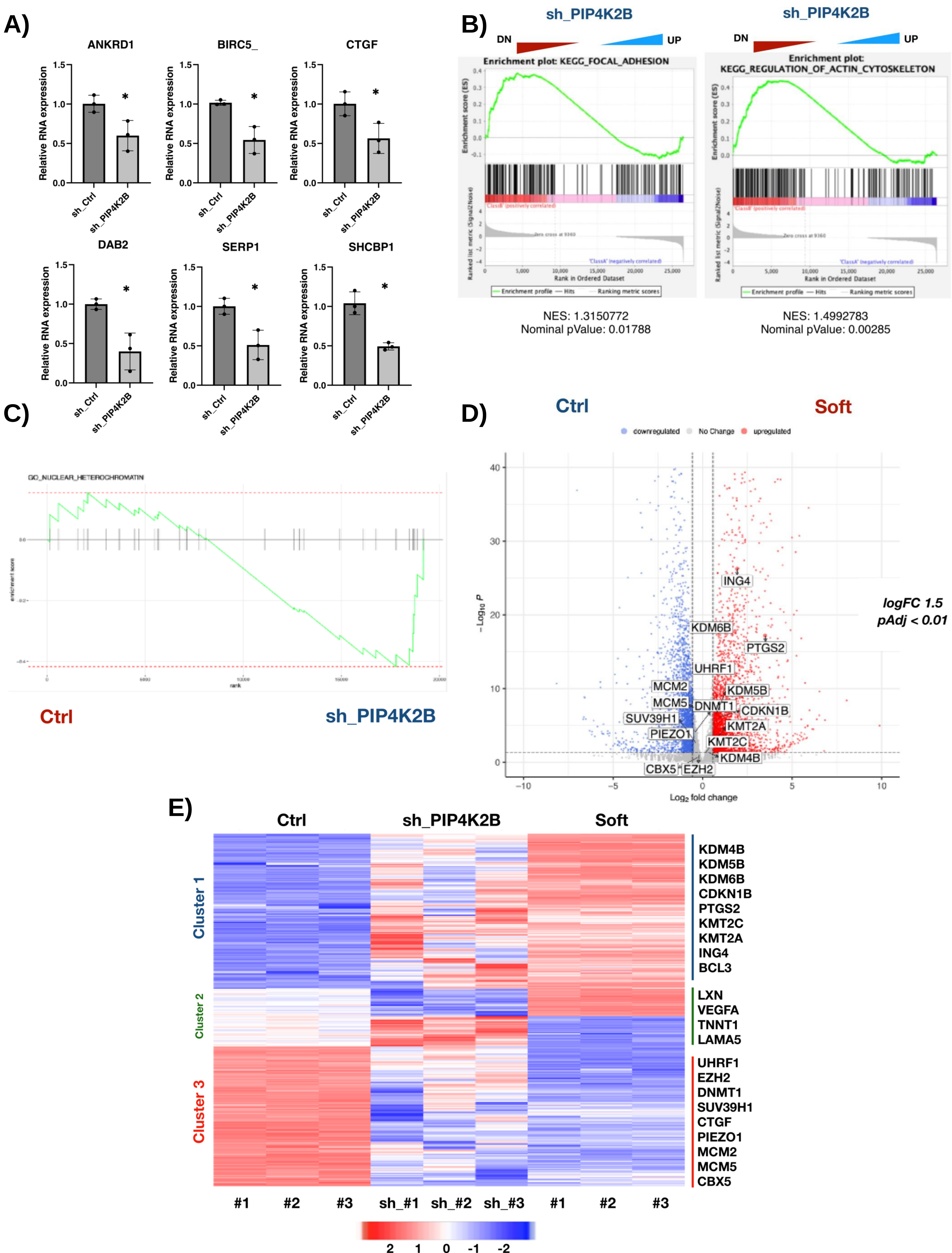
A)
ANKRD1
BIRC5_
CTGF
DAB2
SERP1
SHCBP1
Relative RNA expression
sh_Ctrl
sh_PIP4K2B
B)
sh_PIP4K2B
DN
UP
Enrichment plot: KEGG_FOCAL_ADHESION
Enrichment plot: KEGG_REGULATION_OF_ACTIN_CYTOSKELETON
Enrichment score (ES)
Ranked list metric (Signal2Noise)
Rank in Ordered Dataset
Enrichment profile — Hits — Ranking metric scores
NES: 1.3150772
Nominal pValue: 0.01788
NES: 1.4992783
Nominal pValue: 0.00285
C)
GO_NUCLEAR_HETEROCHROMATIN
enrichment score
rank
Ctrl
sh_PIP4K2B
D)
Ctrl
Soft
downregulated
No Change
upregulated
$-\log_{10} P$
$\log_2$ fold change
logFC 1.5
pAdj < 0.01
ING4
KDM6B
PTGS2
UHRF1
MCM2
KDM5B
MCM5
DNMT1
CDKN1B
SUV39H1
KMT2A
PIEZO1
KMT2C
KDM4B
CBX5
EZH2
E)
Ctrl
sh_PIP4K2B
Soft
Cluster 1
Cluster 2
Cluster 3
KDM4B
KDM5B
KDM6B
CDKN1B
PTGS2
KMT2C
KMT2A
ING4
BCL3
LXN
VEGFA
TNNT1
LAMA5
UHRF1
EZH2
DNMT1
SUV39H1
CTGF
PIEZO1
MCM2
MCM5
CBX5
#1
#2
#3
sh_#1
sh_#2
sh_#3
#1
#2
#3
2
1
0
-1
-2

Supplementary Figure 5. PIP4K2B controls YAP gene signature. A) RT-qPCR analyses of YAP target genes in cells transduced to silence PIP4K2B (sh_PIP4K2B) and control cells (sh_Ctrl). Data are representative of n=3 independent experiments and shown as bar charts +/- standard deviation. Statistical analyses were performed using unpaired two-tailed Student's t test with Welch's correction with p values as *$p < 0.05$ (ANKRD1 pVal 0.0468, BIRC5 pVal 0.0369, CTGF pVal 0.0379, DAB2 pVal 0.0388, SERP1 pVal 0.0267, SHCBP1 pVal 0.0154). B) Gene expression data generated through RNA-seq comparing Ctrl vs sh_PIP4K2B were used for GSEA analyses to extract biological knowledge. GSEA enrichment plots of differentially expressed genes using the KEGG (Kyoto Encyclopedia of Genes and Genomes) Regulation of actin cytoskeleton (hsa04810) and Focal Adhesion (hsa04510). The green curve in the charts corresponds to the enrichment score (ES) curve, which is the running sum of the weighted ES obtained from GSEA software, while the normalized enrichment score (NES), the corresponding one-sided p value are reported within the graph. C) Enrichment plot of GO nuclear heterochromatin terms. The green curve corresponds to the ES (enrichment score) curve, which is the running sum of the weighted enrichment score obtained from fGSEA software. D) Volcano plot representing DEGenes of Soft vs Ctrl conditions. All significantly (pAdj < 0.01, wald test p value adjusted using Benjamini and Hochberg method) deregulated genes are in red (upregulated) and blue (downregulated). Enrichment (log2(fold change)) is plotted on the x-axis and significance (Wald test –log10(p-value two-sided)) is plotted on the y-axis. E) Heatmap of DEGenes found in both sh_PIP4K2B and Soft compared to Ctrl. Genes are presented in 3 clusters: 1) Upregulated genes in both sh_PIP4K2B and Soft; 2) Genes differentially regulated in Soft and sh_PIP4K2B; 3) genes Downregulated in both sh_PIP4K2B and Soft.

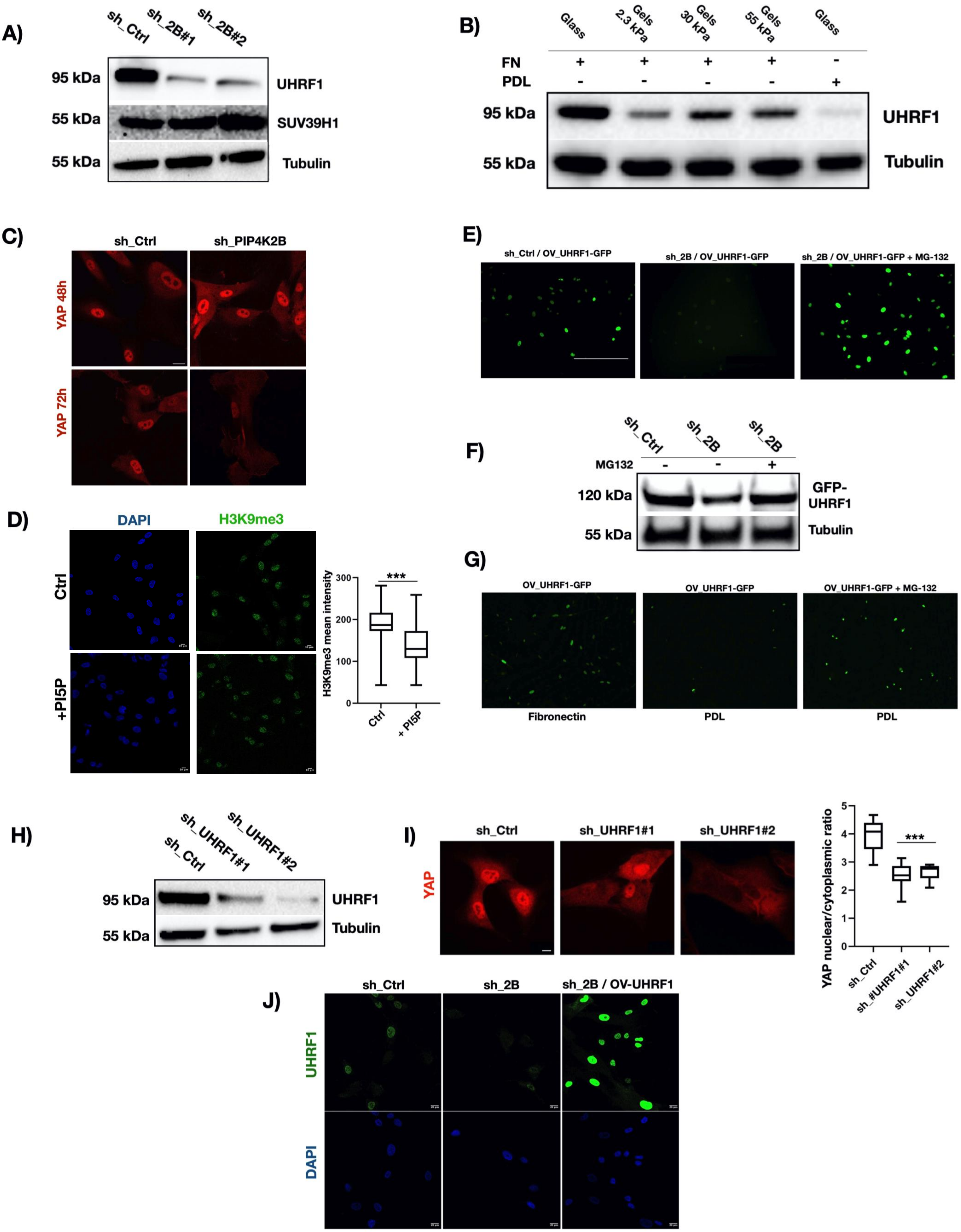

A)
sh_Ctrl
sh_2B#1
sh_2B#2
95 kDa
UHRF1
55 kDa
SUV39H1
55 kDa
Tubulin
B)
Glass
Gels 2.3 kPa
Gels 30 kPa
Gels 55 kPa
Glass
FN + + + + -
PDL - - - - +
95 kDa
UHRF1
55 kDa
Tubulin
C)
sh_Ctrl
sh_PIP4K2B
YAP 48h
YAP 72h
E)
sh_Ctrl / OV_UHRF1-GFP
sh_2B / OV_UHRF1-GFP
sh_2B / OV_UHRF1-GFP + MG-132
F)
sh_Ctrl
sh_2B
sh_2B
MG132 - - +
120 kDa
GFP-UHRF1
55 kDa
Tubulin
D)
DAPI
H3K9me3
Ctrl
+PI5P
H3K9me3 mean intensity
***
Ctrl
+ PI5P
G)
OV_UHRF1-GFP
OV_UHRF1-GFP
OV_UHRF1-GFP + MG-132
Fibronectin
PDL
PDL
H)
sh_Ctrl
sh_UHRF1#1
sh_UHRF1#2
95 kDa
UHRF1
55 kDa
Tubulin
I)
sh_Ctrl
sh_UHRF1#1
sh_UHRF1#2
YAP
YAP nuclear/cytoplasmic ratio
***
sh_Ctrl
sh_#UHRF1#1
sh_UHRF1#2
J)
sh_Ctrl
sh_2B
sh_2B / OV-UHRF1
UHRF1
DAPI

Supplementary Figure 6. PIP4K2B/UHRF1 signalling alters cell mechanics and YAP signalling. A) Western Blotting of hTERT_RPE1 cells transduced to silence PIP4K2B (sh_2B#1/#2), or with empty pLKO_1 vector as control (sh_Ctrl). Immunoblot was performed to detect protein levels of UHRF1 and SUV39H1. Tubulin was used as loading control. B) hTERT_RPE1 cells were cultured for 24 hours on Fibronectin (FN)- or Poly D-lysine (PDL)-coated glass coverslips, and on FN-coated acrylamide gels of different stiffness (2.3 kPa / 30 kPa / 55 kPa), then lysed. Protein lysates were immunoblotted to analyse UHRF1 protein levels. Tubulin was used as loading control. C) Immunofluorescent staining of YAP cellular distribution in sh_Ctrl and sh_PIP4K2B cells related to WB time course experiment presented in Figure 4A (scale bar = 10µm). Quantification of nuclear to cytoplasmic YAP signal ratio are reported. D) Immunofluorescence staining of H3K9me3 and DAPI in nuclei of cells starved overnight and then grown for 4 hours with (+PI5P, pVal < 0.0001) or without PtdIns5P (Ctrl) (scale bar = 20µm). Data quantification is shown as boxplots representing the ratio between the intensity of single fluorescent dots and cell nuclear area (n=3 independent experiments). E)/F) Immunofluorescence and Western Blotting of hTERT_RPE1 cells transduced to silence PIP4K2B (sh_2B) growing with or without MG-132 (5µM), or with empty pLKO_1 vector as control (sh_Ctrl) (scale bar = 400µm). Cells were previously transduced to overxpress GFP tagged-UHRF1. IF to detect GFP-UHRF1 levels and Immunoblot were performed to detect protein levels of exogenous UHRF1. Tubulin was used as loading control. G) Immunofluorescence analysis of GFP-UHRF1 in cells seeded on Fibronectin or PDL (+/- MG-132, 5 µm) coated glass coverslips. H) Western Blotting of hTERT_RPE1 cells transduced to silence UHRF1 (sh_UHRF1#1/#2) or with empty pLKO_1 vector as control (sh_Ctrl). Protein levels of UHRF1 were assessed, using Tubulin as loading control. I) Immunofluorescent staining of YAP cellular distribution in cells treated as in G) (scale bar = 10µm). Quantification of nuclear to cytoplasmic YAP signal ratio are reported (n=2 independent experiments, sh_UHRF1#1 pVal < 0.0001, sh_UHRF1#2 pVal 0.0004). J) Immunofluorescent staining of UHRF1 in cells transduced with empty pLKO_1 vector (sh_Ctrl), with sh_RNAi targeting PIP4K2B (sh_PIP4K2B), and with sh_RNAi targeting PIP4K2B and a plasmid to overexpress UHRF1 (sh_PIP4K2B / OV_UHRF1) (scale bar = 20µm). All immunofluorescence experiments were performed analyzing at least 20 cells, and data shown are representative of at least two independent experiments. Statistical analyses were performed using unpaired two-tailed Student's t test with Welch's correction, with p values as *p < 0.05, **p < 0.01, ***p < 0.001. In boxplots: middle bars are medians, the rectangles span from the first to the third quartiles and bars extent from min to max values.

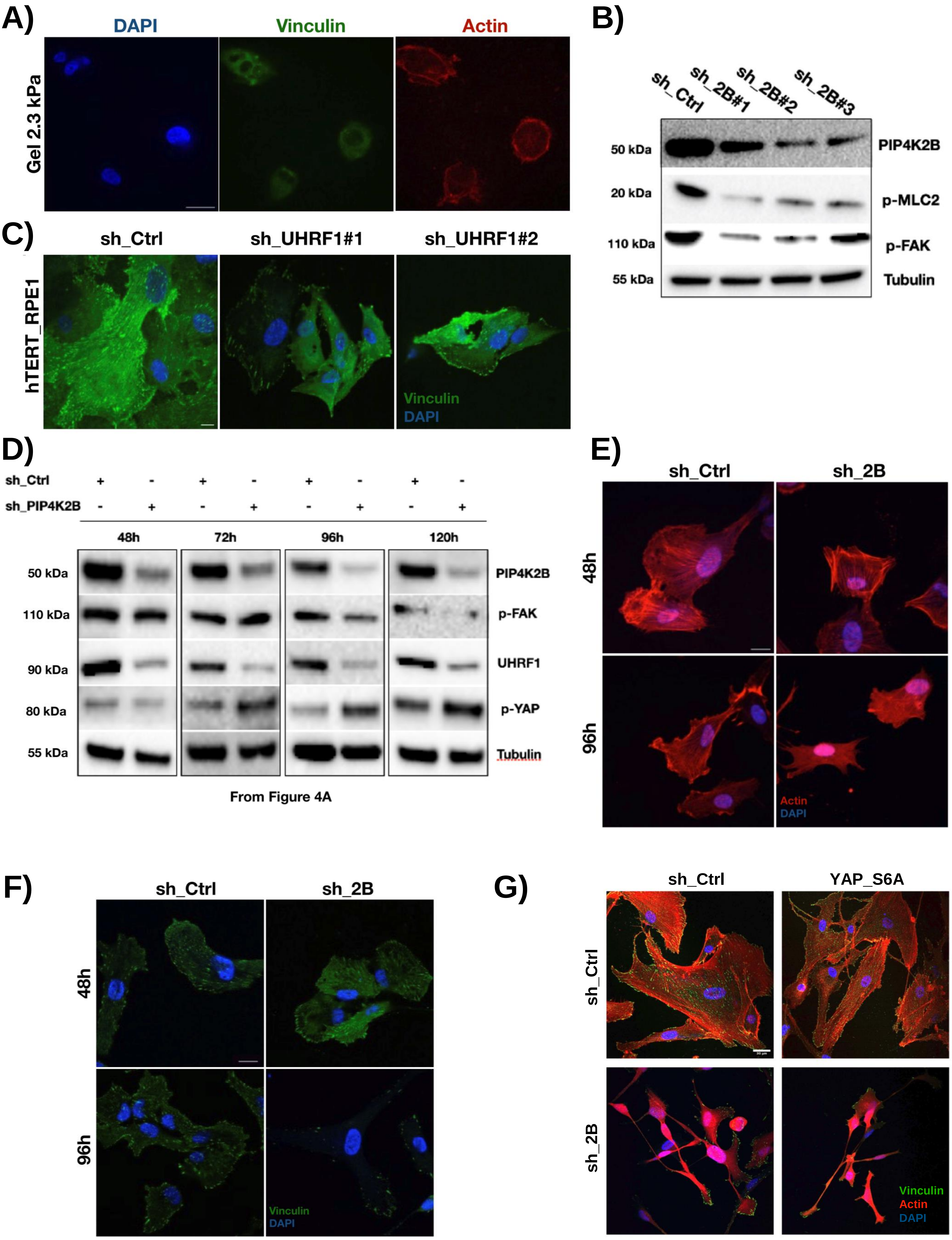
A)
DAPI
Vinculin
Actin
Gel 2.3 kPa
B)
sh_Ctrl
sh_2B#1
sh_2B#2
sh_2B#3
50 kDa
PIP4K2B
20 kDa
p-MLC2
110 kDa
p-FAK
55 kDa
Tubulin
C)
sh_Ctrl
sh_UHRF1#1
sh_UHRF1#2
hTERT_RPE1
Vinculin
DAPI
D)
sh_Ctrl + - + - + - + -
sh_PIP4K2B - + - + - + - +
48h
72h
96h
120h
50 kDa
PIP4K2B
110 kDa
p-FAK
90 kDa
UHRF1
80 kDa
p-YAP
55 kDa
Tubulin
From Figure 4A
E)
sh_Ctrl
sh_2B
48h
96h
Actin
DAPI
F)
sh_Ctrl
sh_2B
48h
96h
Vinculin
DAPI
G)
sh_Ctrl
YAP_S6A
sh_Ctrl
sh_2B
Vinculin
Actin
DAPI

Supplementary Figure 7. UHRF1 depletion phenocopies PIP4K2B silencing. A) Immunofluorescent staining of nuclei (DAPI), Focal Adhesion (Vinculin) and Actin (Phalloidin) in hTERT_RPE1 cells seeded on FN-coated soft surfaces (Gel 2.3 kPa) (scale bar = 20µm). B) Western Blotting analysis of protein levels of protein and phosphorylated proteins in cells depleted for PIP4K2B (sh_2B#1/#2/#3) and control cells (sh_Ctrl). Tubulin was used as loading control. C) Immunofluorescence staining of focal adhesions (Vinculin) in in hTERT_RPE1 cells seeded on FN-coated glass and transduced to silence UHRF1 (sh_UHRF1#1/#2) or with empty pLKO_1 vector as control (sh_Ctrl) (scale bar = 10µm). D) Western Blotting analysis of phosphorylated FAK levels. WB is related to WB shown in Figure 4A. E)/F) Immunofluorescence staining of Actin (Phalloidin) or focal adhesions (Vinculin) in cells treated as in D). Data are related to WB shown in D) (scale bar = 10µm). G) Immunofluorescence staining of focal adhesion in cells transduced to silence PIP4K2B (sh_PIP4K2B) and to concomitantly silence PIP4K2B and overexpress nuclear YAP-S6A. In all immunofluorescence experiments, at least 20 cells were analysed and data reported represent at least 2 independent experiments.

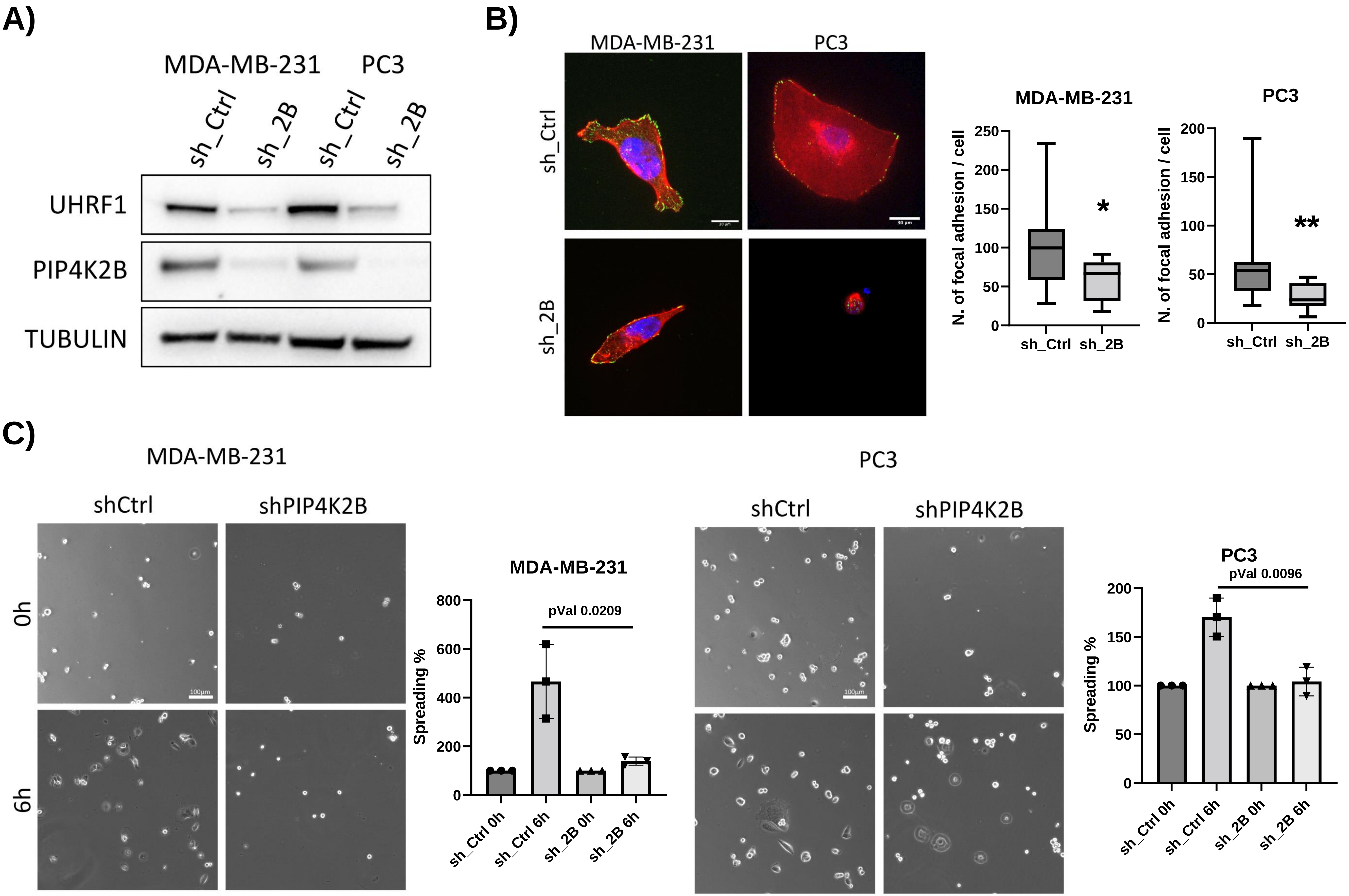
A)
MDA-MB-231
PC3
sh_Ctrl
sh_2B
sh_Ctrl
sh_2B
UHRF1
PIP4K2B
TUBULIN
B)
MDA-MB-231
PC3
sh_Ctrl
sh_2B
MDA-MB-231
PC3
N. of focal adhesion / cell
250
200
150
100
50
0
*
**
sh_Ctrl
sh_2B
C)
MDA-MB-231
shCtrl
shPIP4K2B
0h
6h
100µm
MDA-MB-231
pVal 0.0209
Spreading %
800
600
400
200
0
sh_Ctrl 0h
sh_Ctrl 6h
sh_2B 0h
sh_2B 6h
PC3
shCtrl
shPIP4K2B
PC3
pVal 0.0096
Spreading %
200
150
100
50
0

Supplementary Figure 8. PIP4K2B depletion alters UHRF1 levels and mechanics of cancer cell lines.

A) Western Blot analysis of PIP4K2B and UHRF1 in PC3 and MDA-MB-231 cells transduced to silence PIP4K2B (sh_PIP4K2B). Empty pLKO_1 vector was used as control (sh_Ctrl). B) Immunofluorescence staining of focal adhesion in cells treated as in A). Data are representative of n=2 independent experiments (MDA-MB-231 sh_2B pVal 0.0457, PC3 sh_2B pVal 0.0068). In boxplots: middle bars are medians, the rectangles span from the first to the third quartiles and bars extent from min to max values. C) Spreading assay of cells seeded onto Fibronectin coated coverslips and let spread for up to 6h. Cells were treated as in A) and data are representative of n=2 independent experiments and shown as bar charts +/- standard deviation of comparisons between sh_Ctrl vs sh_2B after 6h of spreading. Statistical analyses were performed using unpaired two-tailed Student's t test, with p values as $*p < 0.05$, $**p < 0.01$.

A)

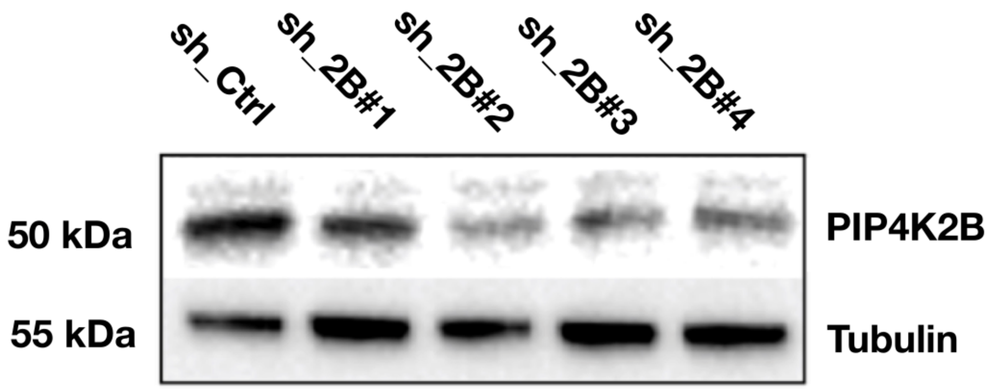
sh_Ctrl
sh_2B#1
sh_2B#2
sh_2B#3
sh_2B#4
50 kDa
55 kDa
PIP4K2B
Tubulin

B)

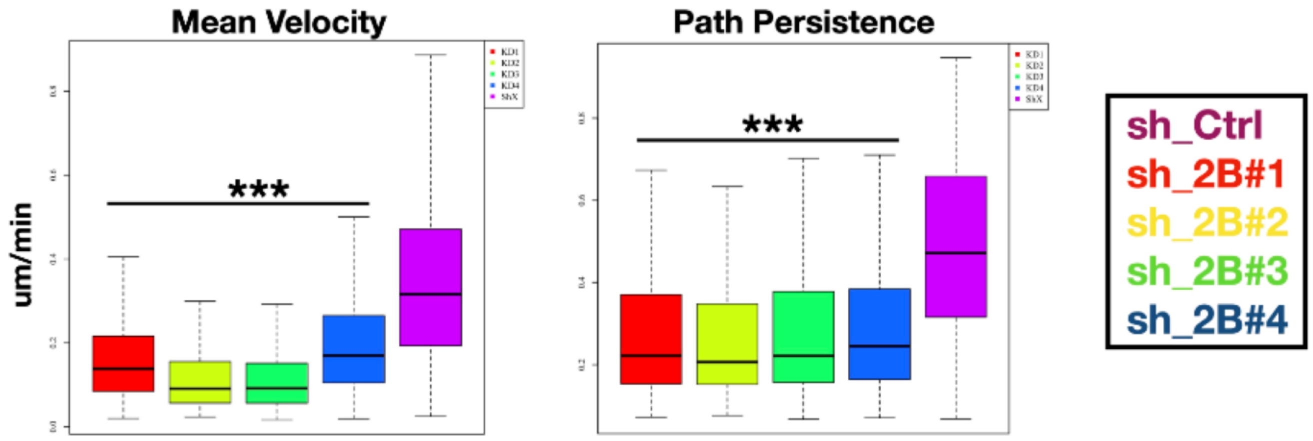
Mean Velocity
Path Persistence
um/min
***
***
sh_Ctrl
sh_2B#1
sh_2B#2
sh_2B#3
sh_2B#4

C)

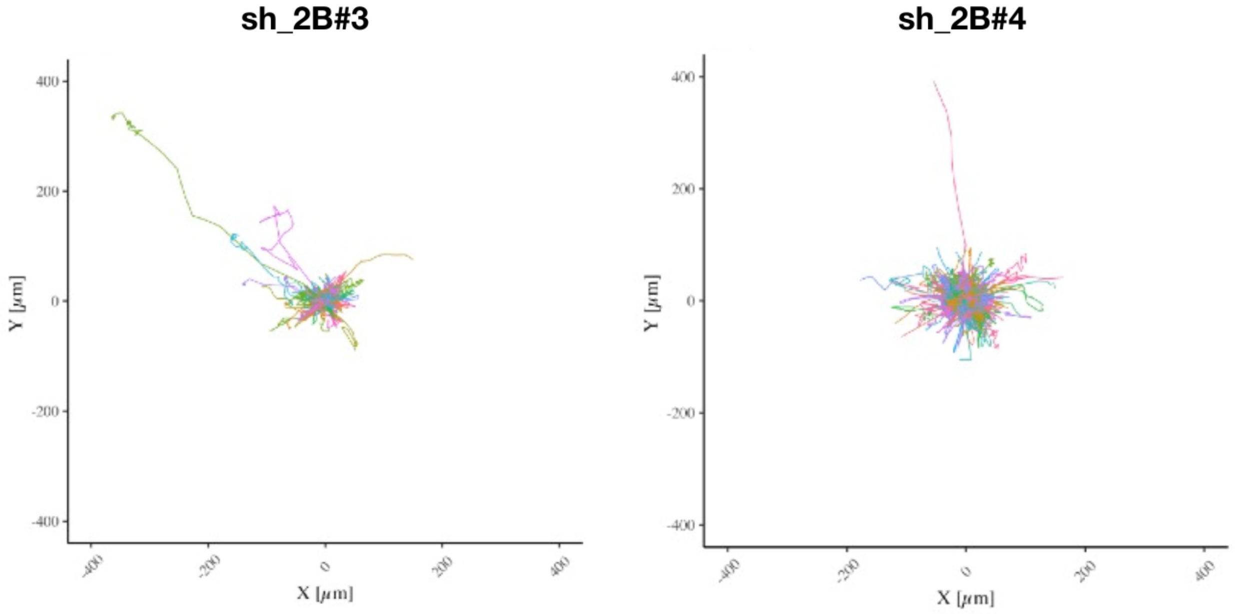
sh_2B#3
sh_2B#4
Y [μm]
X [μm]
400
200
0
-200
-400

Supplementary Figure 9. Depletion of PIP4K2B affects hTERT_RPE1 cell motility. A) Western Blotting analysis of PIP4K2B protein expression in cells transduced with 4 different sh_RNAi targeting PIP4K2B (sh_2B#1/#2/#3/#4). Empty pLKO_1 vector was used as control (sh_Ctrl) and Tubulin as loading control. B) 2D cell motility quantification of cell Mean Velocity and Path Persistence. Cells were treated as in A), and seeded on FN-coated glass coverslips. Nuclei were stained with NucBlue and cell tracking performed for 16 hours. C) Plots representing trajectories of cells depleted for PIP4K2B or UHRF1, and Control cells. Data reported are representative of n=3 independent experiments. Statistical analyses were performed using paired two-tailed Student's t test, with p values as *$p < 0.05$, **$p < 0.01$, ***$p < 0.001$. Box plots: middle bars are medians, the rectangles span from the first to the third quartiles and bars extent from ±1.5*IQR.

Table 1_Hydrogel preparation

Shear Modulus (Pa)	230	34263	55293
40% Acrylamide (mL)	1.25	2.50	2.50
2% Bis-Acrylamide (mL)	0.5	1.88	2.50
H2O (mL)	3.25	0.63	0.00
Volume total (mL)	5	5	5
Stock solution used (Pa)	230	34263	55293
Stock solution volume (uL)	150	300	300
TEMED (uL)	347	197	197
10 % APS (uL)	0.7	0.7	0.7
H2O (uL)	2.5	2.5	2.5
Volume Total (uL)	500	500	500
Final Acrylamide %	3	12	12
Final Bis-Acrylamide %	0.06	0.45	0.6

Table 2_sh_RNAi

Target Gene	ID#number	Sequence
PIP4K2B#1	TRCN0000199907	GCCAAGGACTTGCCAACATTC
PIP4K2B#2	TRCN0000338469	GTTAGGGAGAAGGGTGT
PIP4K2B#3	TRCN0000197129	GAAACCTACATGGTGGTTACC
PIP4K2B#4	TRCN0000196947	GCAAGATCAAGGTGGACAATC
UHRF1#1	TRCN0000004352	CCGCACCAAGGAATGTACCAT
UHRF1#2	TRCN0000004355	TGTGAAATACTGGCCCGAGAA

Table 3_Western Blot Antibodies

Primary Antibodies (WB)
UHRF1, Monoclonal santa Cruz (sc-373750) 1:1000
PIP4K2A (D83C1) monoclonal Cell Signaling #5527 1:1000
PIP4K2B monoclonal Cell Signaling #9696 1:1000
PIP4K2C Polyclonal 17077-1-AP Proteintech 1:1000
beta-Tubulin Monoclonal Santa Cruz sc-5724 1:5000
UHRF1, Monoclonal santa Cruz (sc-373750) 1:1000
SUV39H1 Monoclonal santa Cruz (sc-23961) 1:1000
p-FAK Monoclonal Santa Cruz (sc-374668) 1:1000
p-YAP Ser127 (D9W2I) monoclonal Cell Signaling #4911:1000
p-MLC2 monoclonal Cell Signaling #3674 1:1000
Lamin B1 - Polyclonal ab16048 rabbit Abcam 1:1000
SUN2 EPR6557 Monoclonal ab124916 rabbit Abcam 1:1000
Emerin 4G5 Monoclonal NCL-EMERIN mouse Leica Biosystems 1:5000
Nesprin 1 - Polyclonal HPA019113 rabbit Sigma Life Science 1:500
Nesprin 2 Monoclonal(F-11) Santa Cruz sc-398616 1:1000
GAPDH Monoclonal(0411) Santa Cruz sc-47724 1:1000
SUN1 EPR6554 Monoclonal ab124770 rabbit Abcam 1:1000
Lamin A/C 636 Monoclonal sc7292 mouse Santa Cruz Biotech 1:2000
YAP Monoclonal Santa Cruz (sc-101199) 1:1000
**Secondary Antibodies (WB)**
Anti-Rabbit IgG 1:10000 Promega W4011
Anti-Rabbit IgG 1:10000 Promega W4021

Table 4_Immunofluorescence Antibodies

Primary Antibodies (IF)
Giantin [Golgi marker] PRB-114C Polyclonal PRB-114C rabbit Biolegend 1:500
Giantin [Golgi marker] PRB-114C, Alexa Fluor 488-conjugated Polyclonal PRB-114C rabbit Biolegend 1:1000
Vinculin hVIN-1 Monoclonal V9131 mouse Sigma 1:800
SUN1 EPR6554 Monoclonal ab124770 rabbit Abcam 1:500
Tri-Methyl-Histone H3 (Lys9) H3K9me3 (D4W1U) Rabbit mAb #13969 1:500
YAP Monoclonal Santa Cruz (sc-101199) 1:400
UHRF1, Monoclonal santa Cruz (sc-373750) 1:500
Nesprin 1 - Polyclonal HPA019113 rabbit Sigma Life Science 1:500
Lamin A/C 636 Monoclonal sc7292 mouse Santa Cruz Biotech 1:400
F-actin was visualized using Phalloidin, Fluorescein Isothiocyanate Labeled (Sigma-Aldrich) 1:200
Emerin 4G5 Monoclonal NCL-EMERIN mouse Leica Biosystems 1:1000
PIP4K2B Rabbit doi: 10.1158/0008-5472.CAN-13-0424 1:1000
**Secondary Antibodies (IF)**
Polyclonal Donkey anti-mouse AlexaFluor-488 AB_2340846 (Jackson ImmunoResearch)
Polyclonal Donkey anti-mouse AlexaFluor-594 AB_2340854 (Jackson ImmunoResearch)
Polyclonal Donkey anti-mouse AlexaFluor-Cy3 AB_2340813 (Jackson ImmunoResearch)
Polyclonal Donkey anti-rabbit AlexaFluor-488 AB_2313584 (Jackson ImmunoResearch)
Polyclonal Donkey anti-rabbit AlexaFluor-594 AB_2340621 (Jackson ImmunoResearch)
Polyclonal Donkey anti-rabbit AlexaFluor-Cy3 AB_2307443 (Jackson ImmunoResearch)

Table 5_RT-qPCR probes and primers

Target	ID	Target	FW	RV
CTGF	Hs00170014_m1	PIP4K2B	TGCATGTGGGAGAGGAGAGT	TCTTCAGCTGTGCCAAGAAC
SHCBP1	Hs00226915_m1	GAPDH	CCCCGGTTTCTATAAATTGAGC	CACCTTCCCCATGGTGTCT
ANKRD1	Hs00173317_m1			
SERPINE1	Hs01126606_m1			
DAB2	Hs01120081_m1			
BIRC5	Hs04194392_s1			
GAPDH	Hs02786624_g1			
