# Supplementary material for: PIP4K2B is mechanoresponsive and controls heterochromatin-driven nuclear softening through UHRF1
Source: Nat Commun. 2023 Mar 14;14:1432. doi: 10.1038/s41467-023-37064-0 (PMC10015053; doi:10.1038/s41467-023-37064-0)
Supplement: Supplementary file 2 — Description of Movies [file 41467_2023_37064_MOESM2_ESM.docx]

Description of Additional Supplementary Files

Supplementary Movie 1: Transmitted light live cell imaging of spreading hTERT-RPE1 cells on fibronectin coated glass coverslips. Representative cell for Sh-Control, Sh-PIP4K2B, Sh-UHRF1 and Sh-PIP4K2B with UHRF1 over expression. Time in min, scale bar 50 μm.

Supplementary Movie 2: Transmitted light live cell imaging of spreading hTERT-RPE1 cells on fibronectin coated glass coverslips. Representative cell for Sh-Control, Sh-Control with YAP-S6, Sh-PIP4K2B, Sh-PIP4K2B with YAP-S6. Time in min, scale bar 20 μm.

Supplementary Movie 3: Transmitted light and nuclei tracking of living hTERT-RPE1 cells on fibronectin coated glass coverslips. Representative field of view for Sh-Control, Sh-PIP4K2B#1, Sh-PIP4K2B#2, Sh-PIP4K2B#3 and Sh-PIP4K2B#4. Time in h:min, scale bar 150 μm.

Supplementary Movie 4: Transmitted light and nuclei tracking of living hTERT-RPE1 cells on fibronectin coated glass coverslips. Representative field of view for Sh-Control, Sh-UHRF1#1 and Sh-UHRF1#2. Time in h:min, scale bar 150 μm.

Supplementary Movie 5: Transmitted light and nuclei tracking of living hTERT-RPE1 cells on fibronectin coated glass coverslips. Representative field of view for cells treated with DMSO, A131 [1 μM], A131 [5 μM], THZ [5 μM] and THZ [5 μM]. Time in h:min, scale bar 150 μm.
